# Developing an Assistive Technology for Persons with Mild Cognitive Impairment: A User-Centered Approach

**DOI:** 10.1093/geroni/igaf122.4097

**Published:** 2025-12-31

**Authors:** Lauren Robinet, Liujing Xie, Xin Yao Lin, Raksha Mudar, Walter Boot, Sara Czaja, Neil Charness, Wendy Rogers

**Affiliations:** University of Illinois Urbana-Champaign, Champaign, Illinois, United States; University of Illinois, Urbana-Champaign, Champaign, Illinois, United States; Weill Cornell Medicine, New York, New York, United States; University of Illinois Urbana-Champaign, University of Illinois Urbana-Champaign, Illinois, United States; Weill Cornell Medicine, New York, New York, United States; Weill Cornell Medicine, New York, New York, United States; Weill Cornell Medicine, New York, New York, United States; University of Illinois Urbana-Champaign, Champaign, Illinois, United States

## Abstract

Assistive technology (AT) holds great potential to increase independence for older adults, specifically persons with mild cognitive impairment (PwMCI). MCI affects approximately 20% of individuals aged 50 and above. Despite this prevalence, the existing literature does not include robust information on their attitudes, perceived needs, or desires regarding AT. We conducted six focus groups across two sites (University of Illinois Urbana-Champaign and Weill Cornell Medicine). There were 20 total participants: 6 male, 14 female, aged 60-85, with a TICS-M score between 22-37, indicative of some level of cognitive impairment. The goal of the group discussions was to develop recommendations for an AT system, called SPARC (Supporting Personal Activities and Reinforcing Cognition). The SPARC system offers features designed to aid PwMCI in completing complex activities of daily living, support their cognitive health, increase social engagement, and provide educational opportunities. Discussions focused on content, ease of use, relevance, and integration with other tools. Participants responded favorably to the “all-in-one” nature of the system. They showed some hesitation about potential effort and determination needed to adopt the system. The activity support and education features were well-received. Participants discussed potential barriers to adoption for the social and reminiscence features, most common being relevance to them, ease of use, privacy concerns, and integration with current approaches. These findings underscore the importance of assessing PwMCI’s attitudes and opinions during the early stages of the design process, which will impact the adoption and success of the SPARC or other systems intended to support their needs.

